# Experimental and theoretical evidence of dihydrogen bonds in lithium amidoborane

**DOI:** 10.1038/s41598-020-74654-0

**Published:** 2020-10-15

**Authors:** Ewelina Magos-Palasyuk, Aleksander Litwiniuk, Taras Palasyuk

**Affiliations:** 1 Institute of Physical Chemistry, Polish Academy of Sciences, Kasprzaka 44/52, 01-224 Warsaw, Poland; 2 Faculty of Mathematics and Natural Sciences, Cardinal Stefan Wyszynski University in Warsaw, Woycickiego 1/3, 01-938 Warsaw, Poland

**Keywords:** Applied physics, Chemical physics, Physics, Condensed-matter physics, Phase transitions and critical phenomena, Structure of solids and liquids, Mechanical properties, Energy, Materials chemistry, Physical chemistry, Chemical physics, Energy transfer, Chemistry, Density functional theory, Structure prediction

## Abstract

In situ high-pressure synchrotron X-ray diffraction, Raman scattering, and complementary first-principles calculations have revealed that structural and spectroscopic properties of lithium amidoborane compound are largely determined by multiple heteropolar dihydrogen bonds. The crystal structure of the compound is stabilized by dimeric complexes, wherein molecular ions bind together by intermolecular dihydrogen bonds of unconventional type. This strong intermolecular coupling determines stable character of the crystal structure in the pressure range up to ~ 30 GPa and is spectroscopically manifested by pronounced changes related to molecular vibrations of the amino group: the splitting of stretching modes, the anomalous behavior of wagging modes as well as Fermi resonance due to vibrational coupling of bending and stretching modes, significantly enhanced above 10 GPa. Unconventional nature of dihydrogen bonds is confirmed by the frequency increase, blueshift, of NH stretching modes with pressure. A role of certain hydrogen mediated interactions in the process of dehydrogenation of ammonia borane and its alkali metal derivatives is speculated. Findings presented here call for reconsideration of hydrogen release mechanism from alkali metal ammonia borane derivatives. The work makes significant contribution towards establishing the general theory of ubiquitous and versatile hydrogen mediated interactions.

## Introduction

Fuel cell technologies currently used in automotive applications impose certain requirements on candidate materials for hydrogen storage such as high purity of released hydrogen, favorable kinetics, operation temperature well below 100 °C and ability of spent material regeneration either via on- or off-board procedures. Lithium amidoborane (LiAB), LiNH_2_BH_3_, is regarded as one of the most promising materials for the development of a lightweight storage medium for hydrogen^1^. In general, LiAB is a compound out of a large family of hydrogen-rich Boron–Nitrogen (B–N–H) materials, which number has been growing fast over the last decade^2,3^. When compared to neat ammonia borane, LiAB exhibits remarkable improvement in hydrogen release. The reasons behind the observed differences between ammonia borane and amidoboranes are far from being well understood. There are many indications on direct interaction between different hydrogen species, which facilitates the formation of diatomic hydrogen molecules and significantly improves the overall process of hydrogen desorption from molecular B–N compounds. An outstanding feature about chemical composition of hydrogen dominant B–N compounds is the presence of chemically distinct acidic (H^δ+^) and basic (H^δ−^) hydrogen species resulting from both polar covalent bonds with nitrogen (more electronegative element) and boron, a less electronegative element. While mechanism of hydrogen release from AB seems to be well established^4,5^, possible dehydrogenation pathways in LiAB and from other metal amidoboranes, in general, are a matter of intensive research^6^. The most often cited model the hydrogen desorption mechanism deals with gas phase and posits occurrence of certain transition states, i.e., complexation of MAB monomer, with subsequent metal-assisted hydrogen transfer leading to the formation of the first H_2_ molecule^7–10^. Despite appealing simplicity of the mentioned scenario, several experimental observations raise considerable reservations about its direct implementation to solid phase, namely:

- Unreacted hydrides of respective alkali metals are present already at the beginning of thermal decomposition. This make elucidation of the role of metal hydride hypothetically formed during thermal decomposition less obvious^11,12^;
- Hydrogen release performance of lithium amidoborane–ammonia borane complex LiAB·AB is significantly improved if compared to pure lithium amidoborane^13,14^;
- Liberation of hydrogen from AB is notably enhanced in ionic liquids and in the presence of acids^15,16^;
- Metal-mediated decomposition basically should not depend on the specific crystal structure (no Li diffusion and mass transport of other constituents). In contrast, *α* and *β* phases of LiAB show clearly different parameters of hydrogen desorption^17^;
- The presence of ammonia is claimed in some studies^11,18,19^ while there are studies^13,20^ reporting no emission or very little amounts of ammonia during thermal decomposition of lithium amidoborane;
- No plausible explanation of the endothermic process occurring in material prior to the rapid emission of molecular hydrogen. In the literature it is frequently called a state of melting, however a detailed description of the material structure at this state is lacking^19^.

To the best of our knowledge there is no theoretical study dealing with dehydrogenation mechanism in pure lithium amidoborane in its solid phases. None of the proposed theoretical models for gas phase considers a direct N–H^δ+^–^δ−^H–B interaction as an initial step towards hydrogen liberation. In contrast, first principles calculations revealed the crucial role of direct interactions between hydrogen species belonging to LiAB and AB layers of LiAB·AB compound^14^.

It is obvious, the observed physicochemical behavior is a sum of multiple interactions taking place in materials subjected to certain thermodynamic conditions. In hydrogen-rich substances interactions involving different forms of hydrogen (atom, proton, hydride-ion) play a crucial role in setting up crystal structures and chemical reactivity of these materials. These interactions are very often referred as secondary interactions and among others include hydrogen bonding, heteropolar and homopolar dihydrogen bonding. Limited data available from either experimental or computational research, at this moment, do not allow clear delineation of the fundamental parameters of bonding patterns created by secondary interactions in B–N–H systems.

Here we report the comprehensive experimental and theoretical study that clarifies the bonding properties of α phase of lithium amidoborane. Based on scrupulous analysis of spectral features of major and subtle changes of both geometrical arrangement and dynamics of either lattice or molecular fragments, the study has revealed a strong intermolecular dihydrogen bonding which plays a decisive role in the structural stability of lithium amidoborane. The apparent discrepancy between ambient pressure studies and previous high-pressure report is explained by the unconventional nature of the dihydrogen bonding in this compound.

Previously underestimated or unrecognized dihydrogen bonding should then be taken into consideration towards understanding the mechanism of hydrogen release from lithium amidoborane and other alkali metals ammonia borane derivatives, in general. In this regard, we propose possible directions hydrogen and dihydrogen bonding may affect the process leading to hydrogen desorption.

## Results

There are two crystal polymorphs of lithium amidoborane (LiAB) reported in the literature: *α*-LiAB and *β*-LiAB^21^. In the following part of the manuscript, each time “lithium amidoborane” or “LiAB” is mentioned it should be referred to its *α*-phase unless stated otherwise.

**Figure 1:**
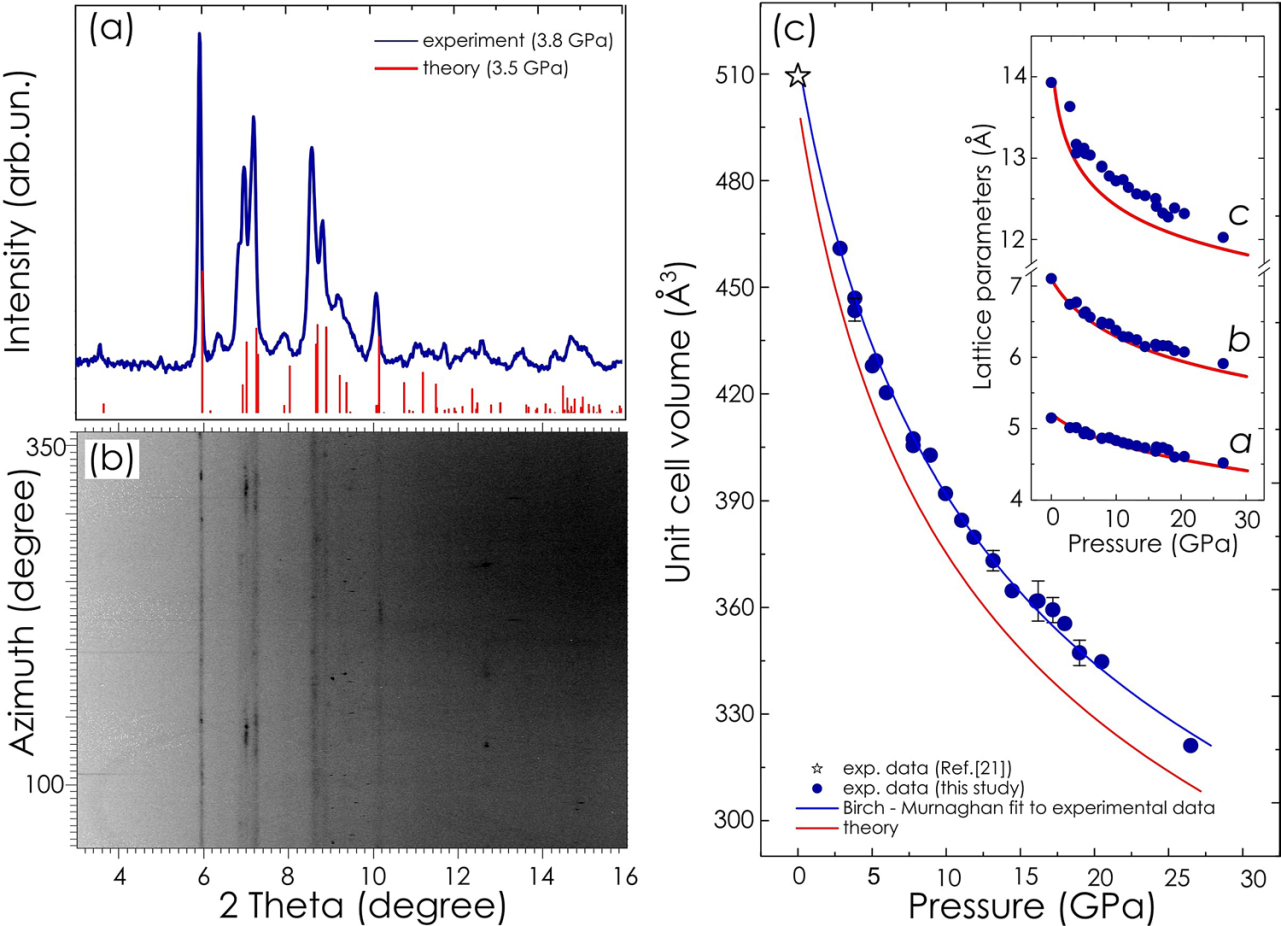
Analysis of X-ray diffraction data related to lithium amidoborane collected at high pressure. (**a**) Representative synchrotron X-ray diffraction pattern of lithium amidoborane acquired at pressure ca. 3.8 GPa (blue line), XRD pattern calculated for pressure value 3.5 GPa (red bars); (**b**) “Cake” integration (intensity versus azimuth) of experimental 2D XRD image acquired at pressure ca. 3.8 GPa; (**c**) unit cell volume as a function of pressure, blue open star—experimental value from Ref.^21^, blue solid symbols—experimental data from this study, blue solid line—fit of Birch–Murnaghan model to experimental data, red solid line—fit of Birch–Murnaghan model to calculated data; (inset) experimental lattice parameters (solid blue symbols) as a function of pressure in comparison to computed data (red lines).

At ambient conditions lithium amidoborane consists of orthorhombic crystals described within Pbca (61) space group with 8 molecules per unit cell^21^. For measurements of X-ray diffraction, a sample of LiAB in form of polycrystalline powder was clamped in a diamond anvil cell at pressure ca. 3.8 GPa, which was an initial pressure of our structural study. To note, 1 GPa is approximately equal to 10,000 atm. The angle resolved X-ray diffraction pattern was consistent with *Pbca* phase, Fig. 1a,b. The angular range (2Θ) of collected data was limited by the diamond cell aperture to ca. 16°, making the refinement of the crystal structure, e.g. atom positions, unreliable. At the same time well resolved diffraction lines (> 12) were clearly indexed within the orthorhombic symmetry and the lattice parameters were accurately determined. Further compression of the sample indicated no sign of significant change in acquired diffraction patterns up to ca. 27 GPa, the highest pressure attained in the XRD measurements. Lattice parameters as well as unit cell volume decreased gradually showing no discontinuity or other indication of major change of the crystal structure, Fig. 1c inset. The orthorhombic *Pbca* structure remained stable revealing a remarkable 38% reduction of the unit cell volume in the pressure range applied in the study.

Similar results related to the stability and compressibility of LiAB were provided by a full geometry optimization of the crystal structure using density functional theory (DFT). Theoretical model of the crystal structure was benchmarked by comparison of computed results to those determined experimentally at ambient pressure^21^. In the range of pressure from 1 atm. to 30 GPa, DFT derived structural models converged to the orthorhombic structure of *Pbca* space group with lattice parameters similar to values determined experimentally. Furthermore, the Birch–Murnaghan model of equation of state^22^ was used to fit experimental as well as theoretically derived data, Fig. 1c.

1 $$P(V)=\frac{3{B}_{0}}{2}\left[{\left(\frac{{V}_{0}}{V}\right)}^\frac{7}{3}- {\left(\frac{{V}_{0}}{V}\right)}^\frac{5}{3}\right]\left\{1+\frac{3}{4}({B}_{0}^{/}- 4)\left[{\left(\frac{{V}_{0}}{V}\right)}^\frac{2}{3}- 1\right]\right\}$$

where B_0_—bulk modulus, B_0_^/^—the first derivative of the bulk modulus with respect to pressure, V_0_—reference volume (corresponding to the ambient pressure).

Resulting values of parameters of equation of state are listed in the Table 1.

**Table 1:** Fitting parameters of the Birch–Murnaghan model.

Data set	V_0,_ Å^3^	B_0_, GPa	B_0_^/^
Experimental	514.6	20.5	3.9
Theoretical	501.9	18.4	3.9

Even though slightly underestimating unit cell volume, in general, the calculations accurately reproduced structural and compressibility characteristics of lithium amidoborane found in our measurements.

Based on the consistency of results obtained experimentally and derived from DFT calculations, the equilibrium structures of LiAB computed at pressure range from 1 atm. to 30 GPa were thus taken for simulation of Raman spectra.

**Figure 2:**
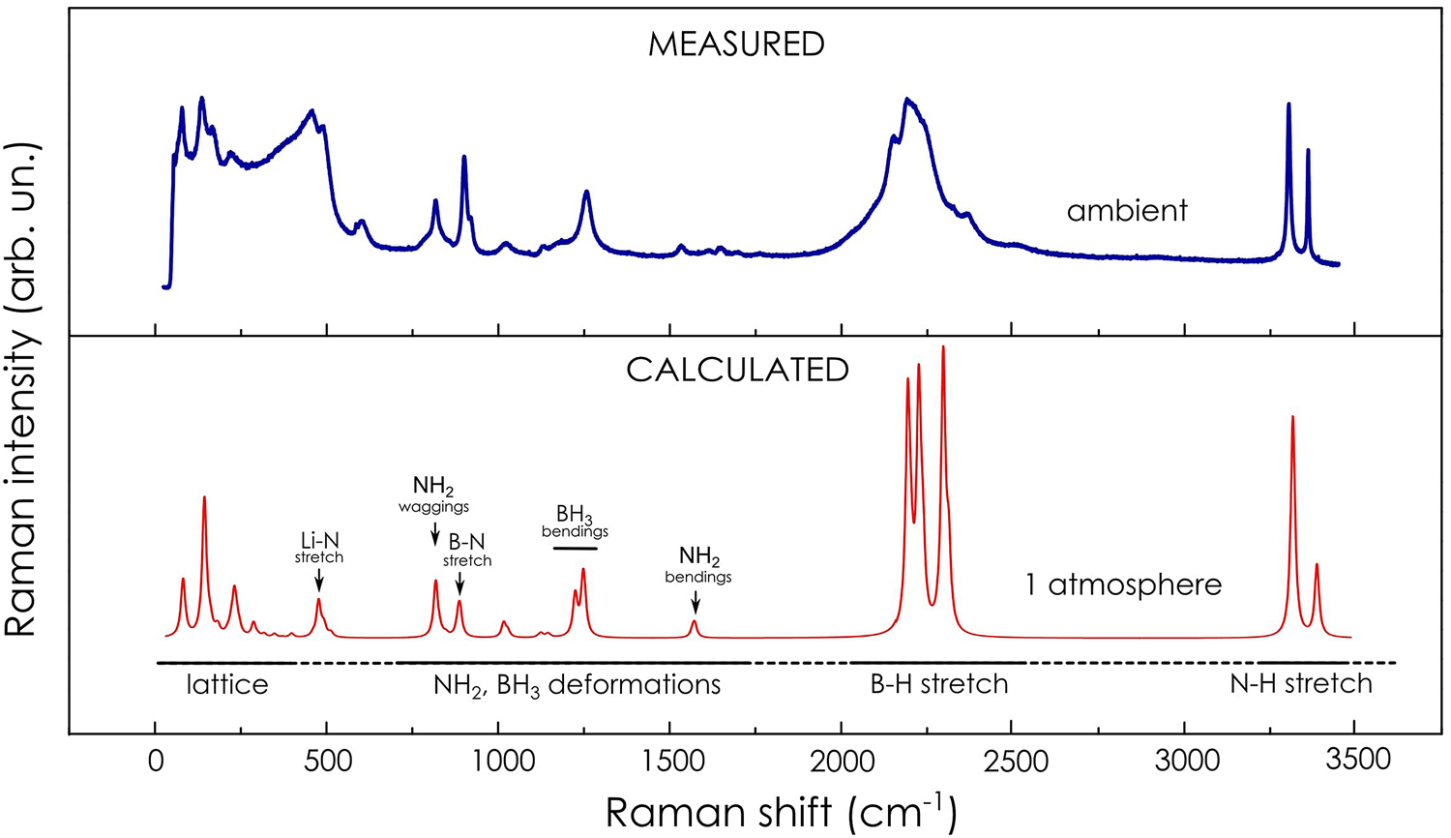
Comparison of Raman spectra of lithium amidoborane collected experimentally and theoretically modelled. (Upper plot) Experimental spectrum acquired from the sample sealed inside glass capillary at ambient pressure. (Lower plot) Raman spectrum lines simulated for pressure 1 atm. Lorents smearing is equal to 15 cm^−1^. Spectral regions and peak assignments are shown for better visualization (crude estimation).

The comparison of the Raman spectrum as calculated by DFT for the pressure of 1 atm. and the experimental spectrum measured at ambient pressure is presented in Fig. 2. The simulated spectrum reproduced experimentally observed spectral features in the whole range of measured frequencies, i.e. ca. 50–3500 cm^−1^. The Raman spectrum consists of several distinct spectral regions related to specific types of vibrational motion: lattice vibrations (< 300 cm^−1^), Li–N stretching (ca. 457 cm^−1^), B–N stretching (ca. 902 cm^−1^), deformations of NH_2_, BH_3_ fragments (wagging, rocking, bending etc. and their combinations) covering frequency range appr. 500–1600 cm^−1^, both symmetrical and asymmetrical stretching of BH_3_ fragments (ca. 2000–2500 cm^−1^) and stretching of NH_2_ fragments (both symmetrical and asymmetrical) in the range of 3300–3360 cm^−1^. It is worth noting, in the Raman spectra collected during high-pressure measurements there were also strong signals from diamond anvil through which the excitation laser beam passed on the way towards the probed sample (for details please see SI). Therefore, for detailed analysis we are addressing primarily spectral regions where signals from the probed sample of lithium amidoborane are not overlapped with the signals of diamond.

**Figure 3:**
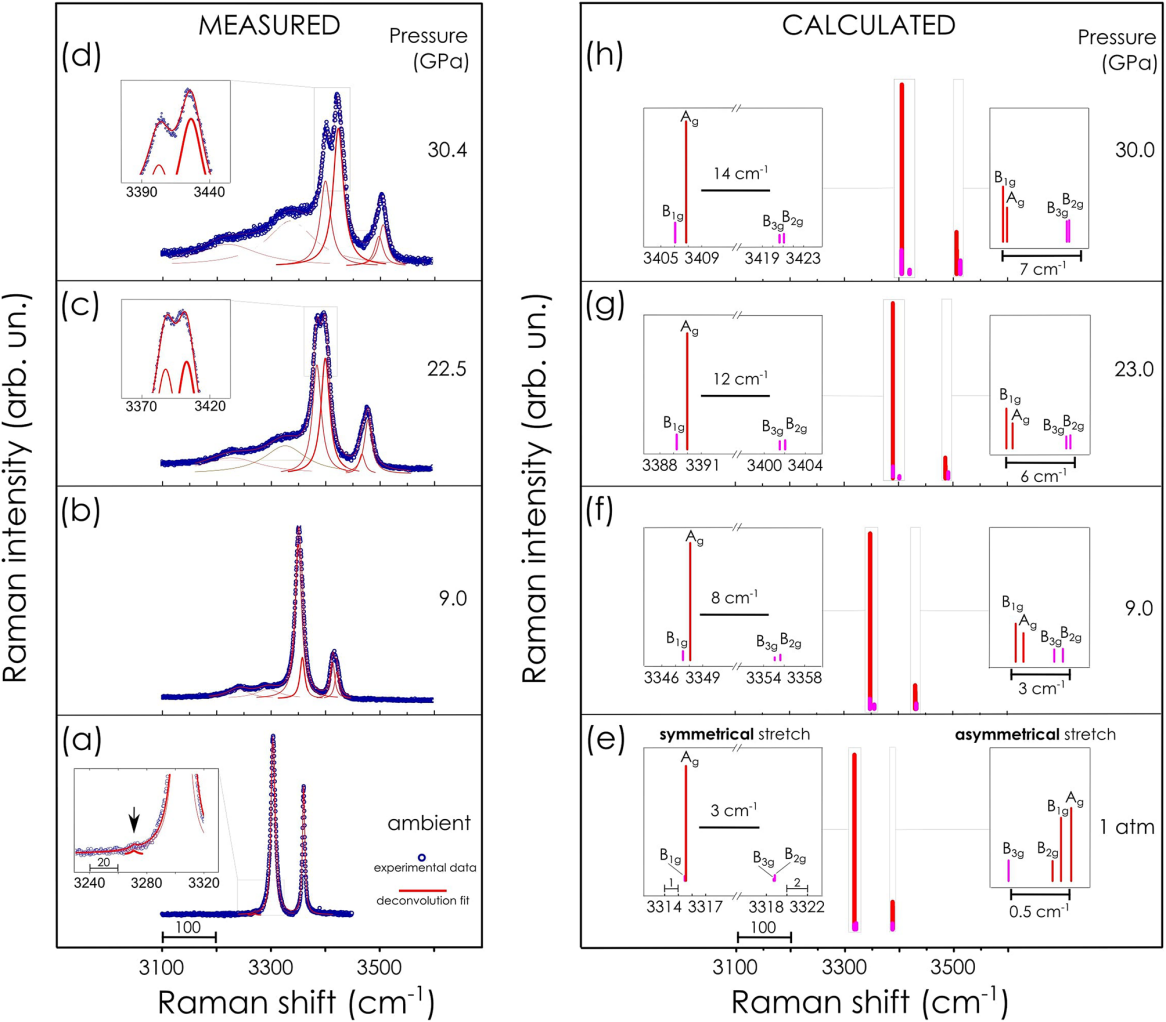
Analysis of Raman spectroscopy data related to N–H stretching vibrations of lithium amidoborane. (Left panel) Representative experimental spectra acquired at ambient pressure, 9.05, 22.5 and 30.4 GPa, plot (**a**), (**b**), (**c**) and (**d**) respectively. Measured signals are presented along with deconvolution analysis. (Inset (**a**)) A signal corresponding to Fermi resonance is shown. (Right panel) Theoretical Raman spectra simulated for selected values of pressure, 1 atm., 9.0, 23.0 and 30.0 GPa, plot (**e**), (**f**), (**g**) and (**h**) respectively. (Insets) Assignment of N–H stretching modes.

The spectral region of N–H stretching vibrations is out of the range of Raman signals originating from diamond anvils therefore any registered signal in this region is attributed to lithium amidoborane. The orthorhombic *Pbca* space group of LiAB contains eight molecules on a point of general (8c) symmetry. Hence each fundamental vibration of the isolated molecule should give rise to 8 factor group modes of the crystal. In contrast, at ambient conditions, spectral region of stretching vibrations of the amino group (–NH_2_) is represented by only two strong signals: a peak of higher intensity centered at about 3307 cm^−1^ (attributed to symmetrical stretching) and the one of lower intensity at about 3365 cm^−1^ (asymmetrical stretching), Fig. 3a. Both signals are well described by a Lorentzian shape. Apparent discrepancy in the number of observed signals and the number of stretching vibration modes predicted by the group theory is most probably due to close energies of some vibrational transitions (degeneracy) resulting in spectral frequencies being distributed in a narrow spectral range.

In this spectral region, there is also a weak signal at position ca. 3270 cm^−1^, Fig. 3a inset. The similar signal was also found in Raman spectra presented in earlier studies reporting results for two different samples, commercially available^23^ as well as synthesized independently for the reported investigation^24^. Origin of this spectroscopic feature was not discussed in the literature, though.

Upon compression, the N–H signals, both symmetrical and asymmetrical stretching, became broader and of asymmetrical shape, which indicated signal splitting into two peaks: of higher and lower intensity, Fig. 3b. That intensity relation within the signal doublets was preserved up to ca. 12 GPa. At higher pressure signal intensity of symmetrical stretching modes exhibited a remarkable redistribution. The signal located at higher frequency showed a steady gain in intensity while initially the most intensive signal, at lower frequency, experienced a considerable attenuation, Fig. 3c,d insets.

In the same range of pressure, a substantial change of yet unassigned signal was detected as well. It evolved into double broad and strong signals of complex spectroscopic structure Fig. 3a–d.

Simulated Raman spectra in the region of N–H stretching vibrations are shown in Fig. 3e–h. The spectrum calculated at ambient pressure is represented by 8 normal modes in agreement with the group theory. Spectral lines are divided into two groups consisting of 4 symmetrical and 4 asymmetrical modes. While there is ca. 70 cm^−1^ separation between two groups in the spectrum, the distribution of frequency values of spectral lines belonging to the particular spectral group falls in a very narrow spectral range: within 3 cm^−1^ (symmetrical modes) and within 0.5 cm^−1^ (asymmetrical modes). Therefore, considering natural width of signals collected at ambient temperature as well as certain instrumental limitations, one should expect strong overlapping of measured signals. Accordingly, in the measured spectrum at ambient pressure, spectral lines are not resolved giving rise only to two signals for particular spectral groups: one signal for symmetrical and the other for asymmetrical band. In overall, calculated spectra reproduced peak positions with high accuracy, within 0.4% and 0.7% for symmetrical and asymmetrical signals, respectively.

**Figure 4:**
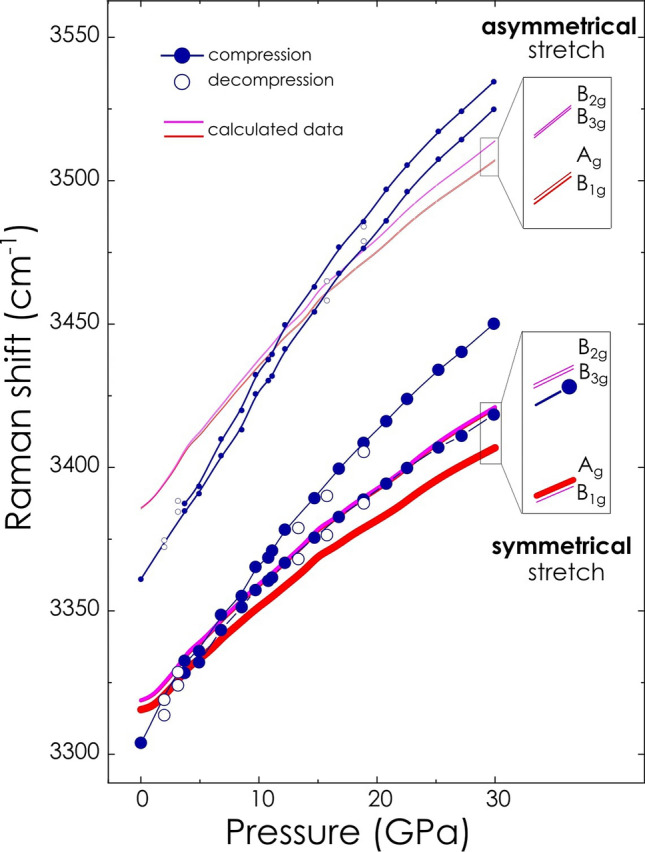
Analysis of Raman spectroscopy data related to N–H stretching vibrations of lithium amidoborane. Frequency of Raman modes as a function of pressure: representative experimental data sets collected during compression and decompression runs are shown by solid blue and empty blue symbols, respectively. Results of DFT calculation are shown by solid red and pink lines. Enlarged fragments of the plot along with mode assignment are shown as insets. Mode assignment corresponding to symmetrical and asymmetrical Raman modes is the same as presented in Fig. 3 (right panel).

At higher pressure calculated spectral lines shift to higher frequencies albeit at different rate. As a consequence of different pressure sensitivity, spectral lines regroup into four distinct doublets: two doublets related to symmetrical stretching modes and two doublets—asymmetrical ones. This behavior resembles the signal splitting of corresponding signals in experimental spectra Fig. 3e–h insets. Separation between symmetrical and asymmetrical stretching modes is gradually increased with pressure Fig. 4.

Intensity relation of corresponding modes, if compared with those in measured spectra, is well reproduced by calculations in the pressure range up to 9 GPa. Instead, the remarkable intensity redistribution detected in the experiment was not reproduced in the calculated spectra above 9 GPa, Fig. 3e–h insets.

Our computations yield no spectroscopic feature in the region of the observed unassigned signal either.

The observed splitting of signals related to N–H stretching well reproduced in our computational results might be an indication of either structural phase transformation or different characteristics (length, strength etc.) of N–H bonds. Considering the continuous reduction of lattice parameters and the unit cell volume we rule out a possible phase transition related to significant modification of the crystal structure. We observed no major change in the spectral region of lattice vibrations, i.e. approximately below 400 cm^−1^, which indicated stable character of the unit cell either up to approx. 30 GPa. Therefore, reasons behind the observed differentiation of N–H stretching vibrations under pressure may stem from changes occurring on a local scale at the molecular level. Characteristics of particular N–H bond oscillators might be affected by the pressure-induced changes of microscopic properties of the compound (e.g., local arrangement of molecular moieties), as well as by specific bonding properties (e.g., noncovalent interactions), which may be manifested by the specific behavior of certain signals in Raman spectra.

**Figure 5:**
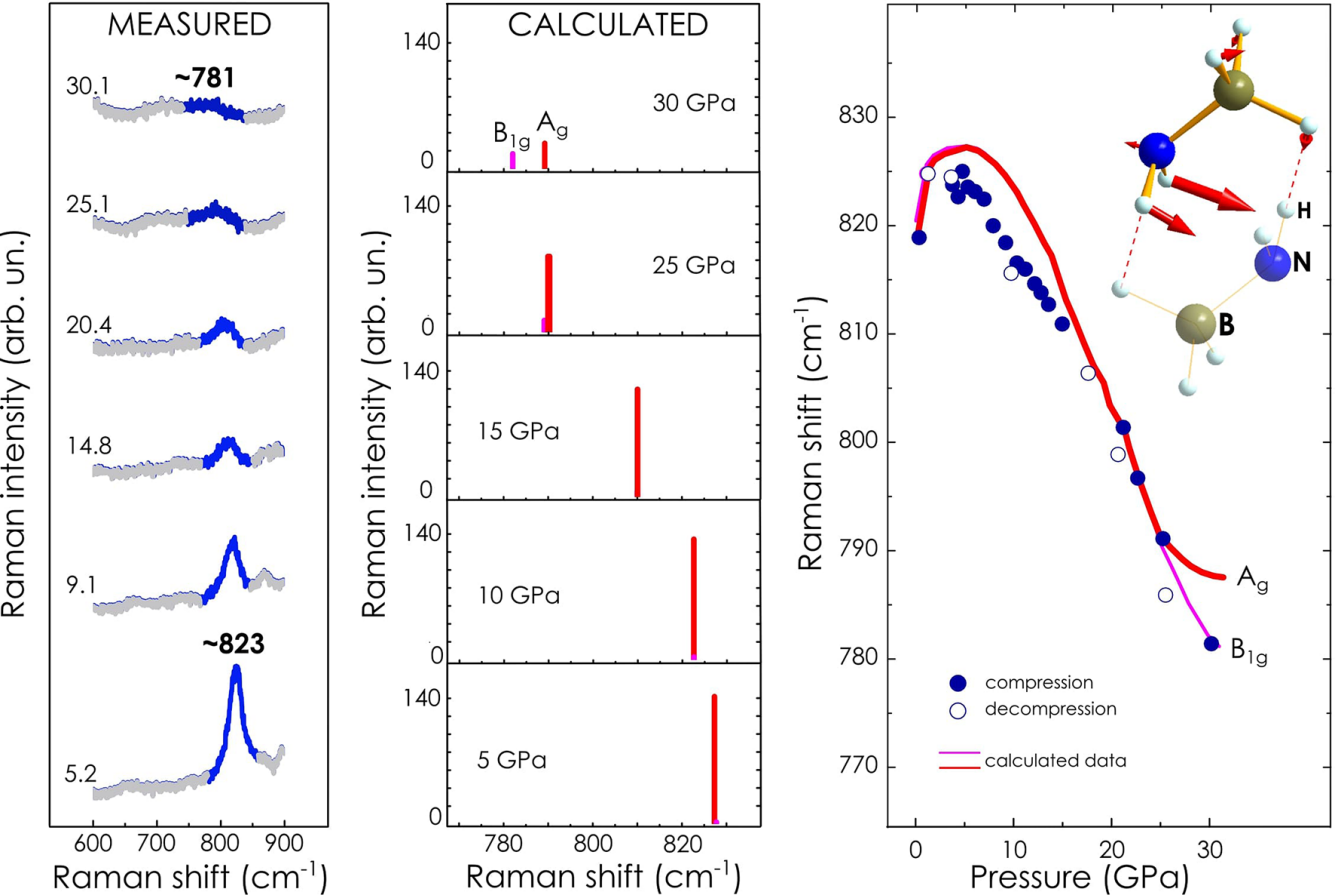
Analysis of Raman spectroscopy data related to N–H wagging vibrations of lithium amidoborane. (Left panel) Representative experimental spectra acquired at selected values pressure of 5.2, 9.1, 14.8, 20.4, 25.1 and 30.1 GPa. Spectra are offset vertically for clarity. A signal at ca. 823 cm^−1^ (at 5.20 GPa) showing a redshift, i.e. frequency decrease, with increasing pressure, is highlighted in blue. (Middle panel) Raman modes theoretically simulated for selected values of pressure 5, 10, 15, 25 and 30 GPa are shown along with mode symmetry assignment. (Right panel) Comparison of frequency data measured experimentally with those calculated. Solid blue symbols depict data collected on compression course while open symbols—on decompression course. (Inset scheme) A schematic illustration of wagging vibration of representative molecular ion; red arrows indicate directions of atom displacements; arrow size is proportional to the amplitude of vibrational motion.

In the ambient-pressure Raman spectrum there is a signal of Raman frequency ca. 820 cm^−1^ which is assigned to a combination of wagging vibrations (inversion motions) of the amino group and deformation vibrations of the borane group, Fig. 5 Right panel inset scheme. It shows an anomalously complex behavior under pressure. In the pressure range up to 5 GPa its Raman frequency slightly increases to ca. 825 cm^−1^, whereas upon further compression the signal shows a pronounced shift to lower values of Raman frequency, a redshift, reaching 781 cm^−1^ at the pressure of 30.1 GPa, Fig. 5 Left panel. It is important to note, the redshift is accompanied by almost a tenfold diminishment of the signal intensity. All the described spectral manifestation related to frequency and intensity of the amino wagging vibrations were accurately reproduced by our DFT calculations Fig. 5 Middle and Right panels. According to calculations, the contribution of -NH_2_ wagging motion to the overall vibration of molecule in this spectral region becomes dominating above 5 GPa. Moreover, atom displacements reveal a significant difference in the amplitude of the motion of particular hydrogen atoms belonging to the amino group, which is illustrated by red arrows of different size, Fig. 5 Right panel inset scheme. These changes in the behavior may result from an additional interaction between one of hydrogen atoms of -NH_2_ group and the other of -BH_3_ group belonging to the neighbor molecule. This additional interatomic interaction acts as an anchor restraining the movement of the one of N–H bonds. One should note that hydrogen atoms involved into the interaction bear different partial charges (H^δ+^ and H^δ−^ from the side of the amino and the borane group, respectively). Hence one may posit the existence of heteropolar dihydrogen bonding between neighboring molecular anions driven basically by electrostatic attraction between proton and hydride species. Since the borane group is a donor of hydride species, the enhancement of the dihydrogen bond under pressure, should impose some restraints on B–H motions as well. This may lead to “freezing out” of certain deformation degrees of freedom which may explain the reducing contribution of the borane group to the overall motion above 5 GPa.

Our earlier studies on potassium and sodium amidoborane (KNH_2_BH_3_, NaNH_2_BH_3_)^25,26^ revealed a significant role of hydrogen bonding determining structural and spectroscopic properties of those monometallic amidoboranes. Data available in the literature on possible hydrogen bonding in lithium amidoborane are rather controversial. On one hand, an investigation performed at ambient pressure^27^ identified plethora of secondary H–H interactions, both heteropolar and homopolar dihydrogen bonds. On the other hand, the high-pressure study^23^ suggested the absence of dihydrogen bonding in lithium amidoborane, which would be also in apparent contrast to pure ammonia borane.

**Figure 6:**
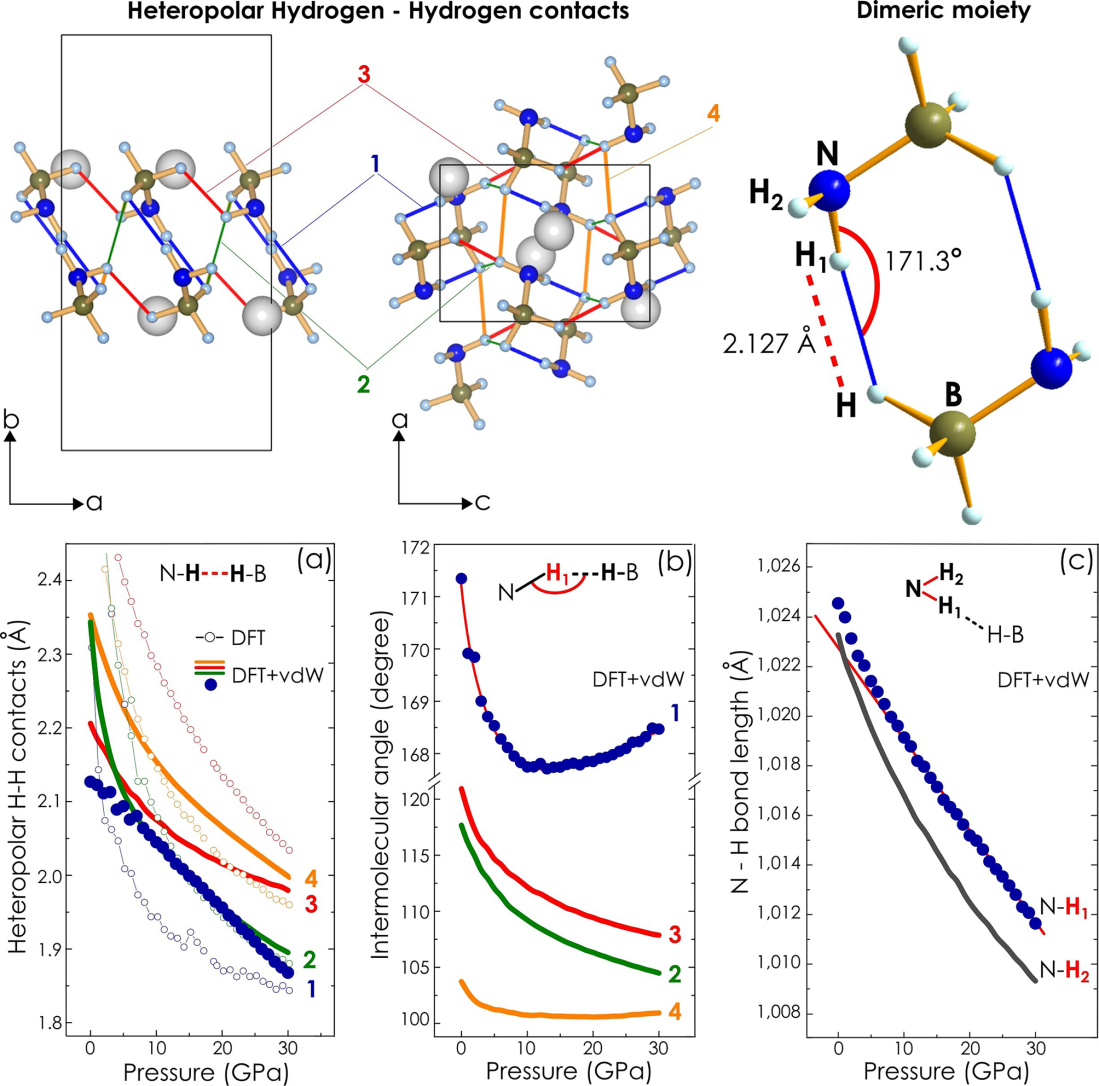
Analysis of geometry optimized crystal structures of lithium amidoborane with particular emphasis on heteropolar H–H (dihydrogen) contacts and N–H bonds. (Upper panel) On the left side and in the middle schematic presentations of the crystal structure at ambient pressure with H–H contacts highlighted in blue **1**, green **2**, red **3** and orange **4** are shown. On the right side a scheme of dimeric complex computed at 1 atm. is presented. (**a**) Evolution of H–H intermolecular distances and intermolecular angles with pressure is presented. Numbering and color coding correspond to those of the upper panel. Data sets for H–H contact derived from models with (DFT + vdW) and without (DFT) dispersion correction are shown by solid lines, solid symbols and empty symbols, respectively. (**b**) Effect of pressure on N–H–H intermolecular angles corresponding to particular dihydrogen contacts are presented. Plotted data sets (solid symbols and lines) are for the model considering dispersion correction. Solid red line presented along with data set highlighted in blue is an eye-guide only. (**c**) Compression behavior of N–H bonds according to the model DFT + vdW. Data set highlighted in blue solid symbols is linearly fit in the pressure range 10–30 GPa.

In search of possible hydrogen bonds, we analyzed geometrical arrangement of molecular fragments, paying particular attention to intermolecular contacts between proton and hydride hydrogen species (H^δ+^–H^δ−^). Geometry criteria described in guidelines of IUPAC community^28,29^ were taken for identifying potential hydrogen bonds, namely, H–H interatomic distances close to 2.4 Å, a threshold value of distance between proton (H^δ+^) and proton acceptor (H^δ−^) as well as hydrogen bond angle above 90°, defined as an angle within donor–proton–acceptor arrangement, i.e. intermolecular angle N–H–H. Into consideration were taken crystal structures fully relaxed using semiempirical correction for van der Waals (vdW) interactions as well. Comparison of results derived from two theoretical approaches, namely, with and without dispersion correction, is presented in Fig. 6a,b. Four H–H contacts meeting geometry requirements for hydrogen bonds were found in the crystal structure. Out of them the one featuring the shortest H–H distance and the N–H–H arrangement close to a linear configuration satisfies the geometry criteria of strong hydrogen bond. Note the opposite tendency in the change of intermolecular hydrogen bond values below 10 GPa and at higher pressure which would suggest the strengthening of the dihydrogen bonding with pressure. Furthermore, this H–H contact links molecules in pairs, dimeric moieties, whereas the others play role of interdimeric linkage. The shortest H–H contact reveals also a remarkable compression behavior, namely, pressure-induced shortening which follows a linear trend. The observed behavior is in apparent contrast to the rest of heteropolar dihydrogen bonds analyzed here.

## Discussion

According to widely recognized and accepted view, the hydrogen bonding is based on a combination of two principal models of interaction: electrostatic and charge-transfer approaches. In case of strong hydrogen acceptors, e.g. N or O atoms possessing electron lone pairs, the latter approach dominates causing charge transfer from the lone pairs to the antibonding orbital of the proton donor. An increased electron density in the antibonding orbitals leads to a weakening of the donor covalent bond which is manifested by bond elongation and a concomitant shift to lower vibration frequency (redshift). This model is not applicable for hydridic species (H^δ−^), which owing to its simpler electronic structure is a weak proton acceptor. Hence stable character of dihydrogen bonding is mainly conditioned by the electrostatic interaction between oppositely charged hydrogen atoms. At the same time, the attraction to H^δ−^ is counterweighted by the attraction from the side of nitrogen donor and by the electrostatic repulsion between nitrogen donor and H^δ−^ acceptor as well. Beside electrostatic interactions, contribution of induction and dispersion energy terms are important to overall stabilization of dihydrogen bonding. It is also worth mentioning, that the presence of electron donating lithium, which considerable increases net charge on nitrogen, and dimerization of NH_2_BH_3_ molecular ions through dihydrogen bonds, may cause significant redistribution of electron density leading to rehybridization of the respective atomic orbitals. An outcome of such a set of forces may be also the contraction of donor bond and associated blueshift of the respective stretching vibration^30,31^.

Experimental results obtained in this study being to a great extent accurately reproduced by relevant DFT calculations provide a firm basis for the conclusion that N–H bonds in lithium amidoborane are not equivalent on account of dihydrogen bonding.

Progressive separation of N–H stretching signals observed in this study is likely a consequence of the strong dihydrogen interaction which involves only one of two hydrogen atoms belonging to the same amino group, Fig. 6 Upper right scheme. The assumption finds confirmation in the analysis of N–H bond lengths. As follows from our computed data, N–H bonds are not equal with respect to interatomic distance between nitrogen and hydrogen. The difference is as high as 0.1% at ambient pressure and increases under pressure, reaching 0.23% at 30 GPa, Fig. 6c. More importantly, N–H bond which is directly involved into dihydrogen bonding is the one with longer interatomic distance which might resemble the weakening of the bond due to the suggested additional interaction. Difference in the length of N–H bonds, increasing with pressure, might be a reason for the enhanced lifting of the degeneracy (energy discrimination) of particular stretching vibrations of amino group in the crystal field leading to the recorded signal splitting.

Dihydrogen bonding should influence the compression behavior of the respective N–H bond. There are many instances of the linear contraction of the covalent bond playing the role of proton donor^25,32^ upon complexation. According to our calculations one of N–H bonds (denoted as N–H_1_, Fig. 6c), which is involved into dihydrogen bonding, shows a linear relationship of bond length shortening above approx. 10 GPa. This correlates with the remarkable linear trend of length contraction of dihydrogen bond identified as a strong one in this study. The other N–H bond, which is not directly involved into the dihydrogen bonding, shows typical behavior under compression. Note that the linear behavior results from calculations taking into account long-range electron correlations that are responsible for van der Waals (dispersive) forces. Different compression behavior means different sensitivity to pressure change, which should also lead to the increasing discrimination of stretching vibration signals.

Because of hydrogen bonding, a partial positive charge on the proton should increase making the respective covalent N–H bond more polar (dipole moment increase) and less polarizable (electron density more localized). As a result, the intensity of corresponding Raman active stretching vibration should decrease. Thus, a significant reduction of the intensity related to the signal of stretching vibration signal acquired at lower frequency may be ascribed to increasing polar character of the N–H_1_ bond, Fig. 3a–d. At the same time the N–H bond not involved into the dihydrogen bonding undergoes undisturbed shortening which means the dipole moment should be reducing (if partial charges on nitrogen and hydrogen remain unaltered) and thus the electron density less localized. Once this N–H bond contributes to intensity of Raman active mode the corresponding signal should exhibit steady increase. In fact, the intensity of the high-frequency signal slightly increases its intensity in the pressure ranges up to ca. 10 GPa. At higher pressure, a unexpectedly much stronger buildup of the intensity takes place. We tentatively assume that this may be due to the redistribution of electron density occurred in molecular moiety on a larger scale as an effect of rehybridization of molecular orbitals of the amino group during pressure-induced strengthening of the dihydrogen bonding. This might lead to increasing contribution of *sp*^2^ configuration and thus additional bond shortening.

The experimentally observed and theoretically reproduced anomalous behavior of the amino wagging vibration modes points to possible enhancement of the dihydrogen bonding above 5 GPa either.

An enhanced mechanical coupling of neighboring molecular ions due to intermolecular dihydrogen bonding should also exhibit certain spectral manifestations. The appearance of the unassigned signal close to N–H stretching modes (ca. 3270 cm^−1^ at ambient pressure spectrum, Fig. 3a inset) we ascribe to the band originating from vibrational mode coupling—Fermi resonance. In the pressure range below 10 GPa, an assumed Fermi resonance should involve symmetrical stretching and overtones of multiple angle bending (scissoring) modes of the amino group, which vibration frequencies lay in spectral region ca. 1540–1650 cm^−1^. Our theoretical calculations deal with harmonic approximation, so characteristics related to overtone band (two photon excitations due to a partial anharmonic character of real vibration) are not reproduced in simulated Raman spectra. Nonetheless, it is possible to correlate changes of fundamental bending modes of amino and borane groups predicted by the theory with the experimentally observed changes of spectral bands in vicinity of signals from symmetrical stretching modes of the amino group. Above 10 GPa multiple bending (rocking) vibrations of borane group may contribute to the resonance excitation as well. Our calculations reveal that with respect to position in Raman spectrum, signals related to bending modes of the borane group are very sensitive to pressure. Above 10 GPa there is an increasing presence of the bending modes of borane group in the region of bending modes of the amino group. This makes plausible a suggestion of additional combination bands resulting from vibrational coupling of borane and amino groups through hydrogen bonding. In solid state, a case of pressure-tuned hydrogen bond mediated resonance interaction of chemically different molecular species was reported for ammonium azide^33^. Intermolecular interaction, like hydrogen bonding, affects the Fermi resonance in condensed phases and leads to a splitting of the optical bands into heavily overlapped broad components. This corresponds to the broad shape of unassigned spectral feature detected in the region of N–H symmetric stretching. In case of hydrogen bonding the frequency might shift somewhat towards higher energy region^34^. The strength of the Fermi resonance depends on the strength of the hydrogen bonds^35^. Pressure-induced increased population of bending modes at relevant spectral region as well as the enhancement of intermolecular hydrogen bonding might be main factors accounting for substantial intensity buildup of the initially very weak signal in the measured spectra under pressure.

## Concluding remarks

Thermodynamic stability and properties of materials depend basically on interatomic interactions. Application of uniform external pressure induces contraction of molecular volume through shortening of interatomic distances and thus significantly altering bond length, bond energies and bond characteristics in general. More importantly, the response of individual type of bonds to compression is different. Therefore, probing materials under variable pressure conditions is potentially of great use in identifying and disentangling complex patterns of chemical bonding.

Results presented in this study explicitly indicate that patterns of chemical bonding of ammonia borane and its derivative, lithium amidoborane, are much alike. Despite introduction of ionic bonding through the replacement of hydrogen atom by lithium element, the resulting material retains an intricate network of heteropolar dihydrogen bonds—secondary interactions mediated mainly by hydrogen species.

Based on observations of bonding schemes of sodium and potassium compounds, NaAB^26^ and KAB^25^, revealed under high-pressure conditions, an alternative hypothesis about the role of hydrogen mediated secondary interactions in the process of thermal decomposition of alkali metal amidoboranes might be tentatively proposed. We now know that in the solid state alkali metal amidoboranes form extended hydrogen-bonded networks presenting either conventional (redshift) or unconventional (blueshift) types of hydrogen bonding. This means hydrogen species under certain thermodynamic conditions may exhibit an increased mobility along hydrogen bonds. It pertains, in particular, to species possessing a proton-like electronic configuration for which quantum nature, e.g. quantum tunneling, contributes significantly. Upon heating, an increased rotation of molecular fragments may promote changes of hydrogen bond networks locally due to either temporary proton hopping or on a global scale as a result of coherent and concerted motions of hydrogen species along hydrogen bonds. In general, as a sequence of proton transfer events taking place in material an increased number of ionic moieties may form which, in turn, due to higher mobility of ions, may induce a plastic-like states as well as the melting of the crystal lattice in whole or in a part (sublattice melting) at certain temperature range. Such hypothetic scenarios may rationalize the existence of the endothermic process prior the decomposition with intensive hydrogen liberation observed during DSC measurements. In the case of potassium amidoborane^36^, which feature redshift hydrogen bonds, the temperature of the endothermic process is clearly lower than that found for sodium compound^37^ featuring blueshift hydrogen bonds. For lithium amidoborane the temperature of the “melting” process is the highest one which could be a result of a different mechanism of proton transfer involving dihydrogen interactions stabilizing the crystal structure.

Therefore, profound knowledge about all possible bonding schemes involving various hydrogen species is essential for understanding dehydrogenation of ammonia borane and its numerous derivatives.

Considering the above-mentioned speculations, molecular dynamics simulations replicating temperature conditions during the thermolysis of lithium amidoborane, performed with particular attention to radial and pair distribution function analysis, could provide key details on evolution of thermally excited states and their effect on the crystal structure at different stages of the dehydrogenation process. Such an approach might provide a valuable insight into dynamics of hydrogen species and changes pertaining to hydrogen bond networks during the endothermic process.

## Materials and methods

### Materials

Sample materials of lithium amidoborane used in this investigation were acquired from two different sources. One of the samples was synthesized from lithium amide, LiNH_2_, and ammonia borane, NH_3_BH_3_, according to a dry mechanochemical procedure using tungsten carbide disk milling vessel with a high energy mill as described in the literature^38^.

Another sample of lithium amidoborane of technical grade was purchased from Sigma-Aldrich and used in an as-received form without further purification.

### X-ray diffraction study

Angle dispersive diffraction patterns of polycrystalline sample were collected at the previous High Pressure Beamline ID 09 of European Synchrotron Radiation Facility (ESRF). X-ray beam of wavelength λ = 0.414552 Å was collimated to a spot 30 × 30 μm^2^ of probed area on the sample. Diffracted radiation was collected by a large area MAR555 flat panel detector. Diamond anvil cell was oscillated during data acquisition in order to get better statistics from randomly oriented grains of the sample. Pressure was determined according to the ruby fluorescence method^39^ using a laser spectrometer setup available at the beamline. Acquired 2D diffraction images were processed using Fit2D software.

### Raman spectroscopy study

Raman scattering measurements were conducted using a confocal layout of a custom designed experimental setup equipped with a Jobin Yvon THR1000 monochromator (focal length 1000 mm, single grating (1200 grooves mm^−1^) providing a resolution of ca. 1 cm^−1^) and a Horiba Synapse CCD detector (thermoelectrically cooled − 75 °C (Peltier effect)). For sample excitation a Melles-Griot He–Ne laser line 632.8 nm was used. Laser beam was focused on the sample to a spot size of less than 3 μm. Measurements were performed in a backscattering geometry. Rayleigh scattering of the laser line was eliminated using a Keiser Optical Systems notch filter.

### DFT calculations

Density functional theory (DFT) calculations were performed using gradient corrected (GGA) exchange correlation Perdew-Burke-Ernzerhof (PBE) functional^40^ and norm-conserving pseudopotentials as implemented in the CASTEP first principles modelling code^41^.

All calculations were carried out at absolute T = 0 K, however Zero Point Energy (ZPE) contributions were not considered in simulations.

Computed results were benchmarked by comparison to data obtained experimentally at ambient conditions: crystal structure solved by Wu et al.^21^, Raman spectrum presented by Ryan et al.^24^ and experimental data obtained in our measurements.

## Supplementary information

Supplementary information.
