# Supplementary material for: Experimental and theoretical evidence of dihydrogen bonds in lithium amidoborane
Source: Sci Rep. 2020 Oct 15;10:17431. doi: 10.1038/s41598-020-74654-0 (PMC7567059; doi:10.1038/s41598-020-74654-0)
Supplement: Supplementary file 1 — Supplementary information. [file 41598_2020_74654_MOESM1_ESM.pdf]

SUPPLEMENTARY INFORMATION

EXPERIMENTAL AND THEORETICAL EVIDENCE OF *DI*HYDROGEN BONDS IN LITHIUM AMIDOBORANE

Ewelina Magos-Palasyuk[1], Aleksander Litwiniuk[1], Taras Palasyuk[2,*],

[1] Institute of Physical Chemistry Polish Academy of Sciences, Kasprzaka 44/52, 01 – 224 Warsaw, Poland

[2] Cardinal Stefan Wyszynski University in Warsaw, Dewajtis 5, 01 – 815 Warsaw, Poland

* corresponding author

METHODS (detailed description)

Details of experimental measurements.

Raman signals related to diamond are present as a very strong signal (saturating CCD detector) at Raman frequency 1334 cm^{-1} (first order Raman) and a broad signal of an irregular shape (second order Raman) covering Raman frequency range from ca. 2000 – 2700 cm^{-1}. The former of the signals overlaps with signals related to bending vibrations of the -BH_3 group whereas th latter signal heavily overlaps with signals originating from B – H stretching vibrations of lithium amidoborane. At higher pressure the 1st order Raman signal experienced considerable and gradual broadening which reflected certain pressure distribution in the bulk of the diamond anvil as a response to external force application. At pressure 30 GPa the strong signal of diamond covered Raman frequency range from 1334 – 1390 cm^{-1}. If 2nd order Raman signal of diamond is concerned, no considerable broadening was detected however it its shape profile might have been changed at higher pressure.

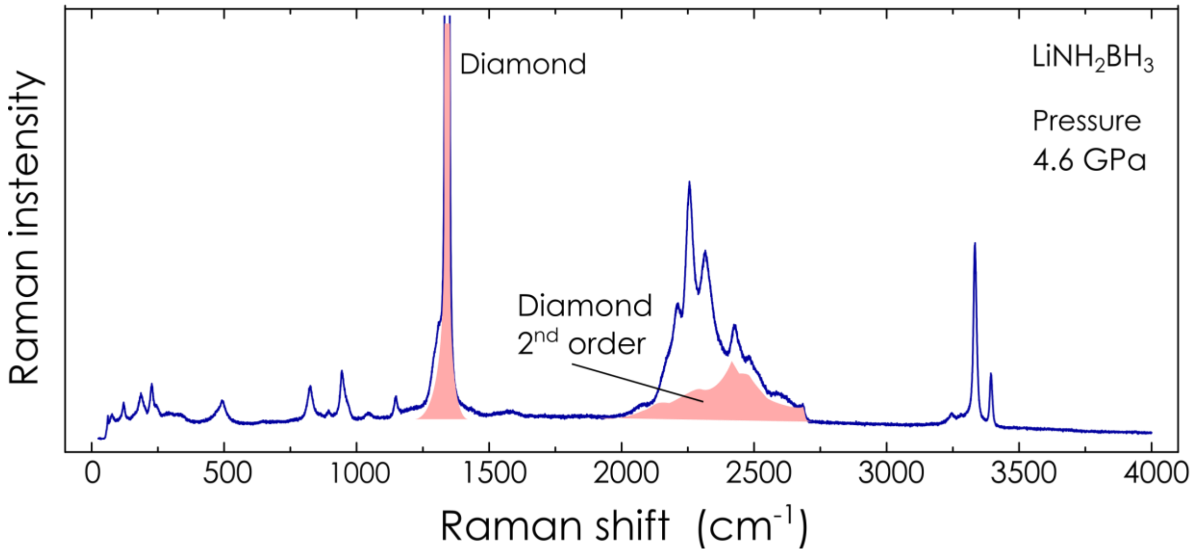

Figure S1. Representative Raman spectrum of lithium amidoborane acquired from the sample in diamond anvil cell. Signals from diamond are highlighted in red (crude estimation).

Both samples have been studied by Raman spectroscopy collecting data during three independent compression runs up to ca. 30 GPa and subsequent decompression to ambient pressure. Spectroscopic data collected from samples of different sources showed high degree of consistency. The sample synthesized directly for this study has been used for X-ray diffraction measurements in a single pressure run up to 27 GPa.

Diamond cells used for high-pressure measurements were equipped with type-I diamond anvils of conical design (Boehler – Almax) [1] with flat culets of 500 μm size. Gaskets made of stainless steel pre-compressed to ca. 30 – 40 μm thickness with a hole of ca. 200 μm in diameter (laser drilled out in the center of the indentation) were used as a sample chamber. Sample in form of powder was loaded under dry nitrogen atmosphere in glovebox and its homogeneity was confirmed by Raman spectra taken from different regions of the sample. No admixture or traces of hydrolysis products were detected in Raman spectra either. Ruby spheres (3 to 5 pcs) of several micrometer in diameter were loaded together with the sample and were used as in situ pressure gauge. Except one pressure run, no pressure transmitting medium was used in the sample chamber, because sample material is fairly soft. For comparison, one run of spectroscopic measurements was performed with mineral oil as a pressure medium. No noticeable difference was observed in the collected spectra if compared to measurements performed without pressure medium.

Details of DFT calculations.

A plane-wave basis set with energy cutoff of 770 eV was employed. Self-consistent field tolerance of 5.0×10^{-7} eV/atom was chosen.

Geometry optimization, including refinement of atomic coordinates and lattice parameters, was performed until the following parameters were converged below defined convergences tolerances (for two successive iterations): total energy change smaller than 5×10^{-6} eV/atom, maximum force per atom below 0.01 eV/Å, pressure smaller than 0.02 GPa, and maximum atomic displacement not exceeding 5×10^{-4}Å. For geometry optimization at certain hydrostatic pressure the Broyden-Fletcher-Goldfarb-Shanno (BFGS) method was used. The quality of this basis set was kept fixed as the unit cell volume varied during geometry optimization.

The normal-mode vibrational analysis was performed using linear response method within the frame of density-functional perturbation theory (DFPT). Calculations were performed using again the PBE functional and norm-conserving pseudopotentials. Phonons were calculated within quasi-harmonic approximation. Brillouin-zone integration was performed according to the Monkhorst-Pack method [2] with a sufficiently dense mesh of k-points to yield accurate convergence. Raman intensities were calculated using the formalism presented in the literature [3, 4].

Moreover, the effect of dispersion interactions, generally termed as van der Waals (vdW), was studied using a semi-empirical dispersion correction scheme proposed by Grimme [5] as implemented in CASTEP.

SUPPLEMENTARY REFERENCES

1. Boehler, R. & De Hantsetters, K. New anvil designs in diamond-cells. *High Press. Res.* **24,** 391–396 (2004).
2. Monkhorst, H. J. & Pack, J. D. Special points for Brillouin-zone integrations. *Phys. Rev. B* **13**, 5188 – 5192 (1976).
3. Porezag, D. & Pederson, M. R. Infrared intensities and Raman-scattering activities within density-functional theory. *Phys. Rev. B* **54**, 7830 (1996).
4. Gonze, X. & Lee, C. Dynamical matrices, Born effective charges, dielectric permittivity tensors, and interatomic force constants from density-functional perturbation theory. *Phys. Rev. B* **55**, 10355 (1997).
5. Grimme, S. Semiempirical GGA-type density functional constructed with a long-range dispersion correction. *J. Comput. Chem.* **27**, 1787 (2006).
